# Is aging “normal”?

**Published:** 2022-12-29

**Authors:** Chloe Johnson, Warren Ladiges

**Affiliations:** a Department of Comparative Medicine, School of Medicine, University of Washington, Seattle, WA 98195, USA.

**Keywords:** Aging, “Normal” aging, age-related changes, geropathology, resilient aging

## Abstract

The descriptive term “normal” aging is often used in scientific literature to indicate commonly occurring changes with increasing age in the absence of overt disease. However, significant molecular and geropathological changes are increasingly present to indicate there is nothing normal about aging. Thus, the term “normal” aging is scientifically incorrect. There are changes in multiple genetic and epigenetic processes and pathways that drive aging, and some individuals are more resilient to these changes than others. Thus, “resilient” aging would be a more correct term to represent a major emphasis on investigating mechanisms and therapeutic targets for resilience, rather than a label of “normal” aging that is misleading and currently receives relatively little attention.

Everyone increases in chronological age once a year, which is considered a normal event and celebrated (or not) on a regular basis. But is there such a thing as normal aging? Normal aging is a descriptive term used frequently in published scientific literature to indicate processes and pathways that similarly change with increasing age in a majority of the population in the absence of overt disease. However, if we take a look beneath the surface, deep into pathological changes that occur in cells with increasing age, nothing appears normal. And in fact, changes become more abnormal with increasing chronological age. So-called “normal” histological changes are considered lesions because histologically they are different from the histology seen at younger ages. Is there such a thing as a normal lesion? We think not, even though many pathologists view the presence of age-related lesions as a normal occurrence for older age groups.

An age-related lesion is considered abnormal because it is not normal in the true sense of geropathology, even though it may not be related to any type of overt disease [1]. It is still a lesion and still not normal. The development of age-related lesions occurs as the result of changes in gene function over time that challenges the way cells can operate, and forces them to adapt. With this adaptation, cells respond in any number of ways depending on their specific role in a tissue milieu. Fibroblasts, for example, respond to develop fibrosis, which in the majority of cases is mild, intermittent, and highly localized. These types of mild age-related lesions are not normal, but yet not generally associated with a disruption in the overall function of a particular organ.

The point of this brief discourse is to provide a convincing argument that the term “normal aging” should not be used because it is scientifically incorrect. Aging consists of abnormal changes that occur over time and in varying degrees in every living creature. In human aging, we know that some individuals are more resilient, so maintain a physically and mentally fit condition with increasing age, while others are less resilient and become increasingly compromised with increasing age [2]. There is thus a tendency to label resilience to aging as normal aging and lack of resilience as abnormal aging. Again, this description lacks scientific merit because changes are still occurring in both resilient and non-resilient groups, but in relative degrees.

We have evidence of the relative nature of resilience to aging in one of our mouse models using brain aging as an example. We recently described naturally occurring age-related cognitive impairment (ARCI) in middle-aged C57BL/6 mice [3]. Interestingly, only about half of the mice tested showed strong evidence of cognitive impairment. The other half was only mildly impaired, but still not normal compared to younger-aged mice. Preliminary observations with molecular and transcriptomic profiling suggested relative differences between resilient and non-resilient brain aging groups in several aging pathways compared to the young mouse group. The point is that brain aging, and aging in general is not a progression of normalcy with increasing age. It is a pathway of abnormal changes that gradually increase in intensity in all of us, yet some are fortunate to have levels of innate resilience and appear to be relatively healthy at an old age, while others are less fortunate and are faced with varying degrees of compromised living and age-related disease at old age.

So, to answer our question- Is aging “normal”? That would be an emphatic NO. There are changes in multiple processes and pathways that drive aging, and as we have emphasized, some individuals are better equipped to handle these changes, at least for a period of time, than others. Thus, the concept of “resilient” aging would be much more productive in investigating mechanisms and therapeutic targets than the label of “normal” aging which currently receives relatively little attention.
